# “Learning to Live Without Him in My Home When a Piece of My Heart Went with Him”: End-of-Life Decision-Making Experiences of Companion Animal Caregivers

**DOI:** 10.3390/ani16182890

**Published:** 2026-09-14

**Authors:** Lori R. Kogan, Jennifer Currin-McCulloch, Sohaila Jafarian, Emma Klein, Kathleen Cooney

**Affiliations:** 1 Department of Clinical Sciences, Colorado State University, Fort Collins, CO 80521, USA; 2 School of Social Work, Colorado State University, Fort Collins, CO 80523, USA; jen.currin-mcculloch@colostate.edu (J.C.-M.); sohaila.jafarian@colostate.edu (S.J.); 3 School of Social Work, Columbia University, New York, NY 10027, USA; ebrown49@uoguelph.ca; 4 Companion Animal Euthanasia Training Academy, Lutz, FL 33549, USA; cooneydvm@gmail.com

**Keywords:** human–animal bond, end-of-life care, euthanasia, guilt, meaning making

## Abstract

As companion animals approach end of life, their caregivers often face complex decisions that require balancing their pet’s quality of life, relief from suffering, potential medical interventions, financial and resource barriers, and the difficult question of whether and when to pursue euthanasia. The purpose of this qualitative study was to explore how companion animal caregivers experience end-of-life decision-making for their pet and the care they received from their veterinary team. The study examined the experiences of 143 pet caregivers who responded to an online survey between September and December 2025. Data collection included caregiver’s responses to four open-ended prompts inquiring about their experiences with their pet’s end-of-life. Questions explored what they regarded as the most challenging and meaningful aspects, regrets they held about their pet’s death, and advice for veterinarians about how best to support pet owners in their pet’s final moments. Data were analyzed using Braun and Clarke’s reflexive thematic analysis. Results highlighted caregivers’ distress surrounding decisions about whether and when they should pursue euthanasia. Caregivers described balancing multiple considerations in their decision-making, including their pet’s overall welfare, the needs and perspectives of family members, resource constraints, access to specialized veterinary care, and their own caregiving fatigue. Their responses demonstrated grief, regret, guilt, and meaning making. Veterinarians’ compassion, transparency, and forward-thinking enabled caregivers to plan for rituals and meaningful death experiences for their pets and their families. Suggestions for veterinary care emphasized the importance of relationship-centered care in addition to technical expertise. Veterinary end-of-life care should prioritize empathy, early and transparent communication, and opportunities for cherished time during the pet’s final moments.

## Simple Summary

The purpose of this qualitative study was to examine how companion animal caregivers experience end-of-life decision-making. Data were collected through open-ended questions in an online survey and analyzed using reflexive thematic analysis. Findings revealed caregivers’ struggles in navigating the tensions of wanting to provide the most compassionate care to their pets, while holding onto their pet as long as possible. Participants reflected on meaningful moments around the end-of-life, as well as guilt and regret they held about the way their pet died. They also highlighted the importance of positive encounters with veterinary teams that enabled intentional end-of-life rituals and opportunities for enhanced communication and empathy from providers. Participants emphasized the need for proactive tailored communication that acknowledges caregivers’ preferences, access to resources, and honors the human–animal bond. These findings suggest that relationship-centered animal end-of-life care that entails informative, empathic, and skilled care can ease caregivers’ uncertainty and responsibility surrounding when and how to say goodbye to their companion animal.

## 1. Introduction

Advances in preventive care, diagnostics, chronic disease management, and specialty medicine have greatly extended the lifespan of companion animals [1,2,3,4]. As dogs and cats are increasingly living into old age, more families are experiencing prolonged periods of caregiving during progressive or terminal illness. End-of-life (EOL) care has therefore become an increasingly important aspect of companion animal veterinary practice, encompassing not only medical management but also complex emotional, ethical, and practical challenges for caregivers [5,6,7].

For many pet caregivers, the final stage of a companion animal’s life involves balancing competing priorities that include preserving quality of life, minimizing suffering, considering treatment options, managing financial and logistical constraints, and determining if, and when, euthanasia is appropriate [8,9,10,11]. These decisions are often made within the context of a profound bond and are frequently accompanied by anticipatory grief, uncertainty, caregiver burden, guilt, and concerns about making the right decision at the right time [5,9,12], which even veterinarians struggle with when faced with the loss of their own pet [13]. Similar to family caregiving in human medicine, caring for a seriously ill companion animal can provide meaning and purpose, yet can also create considerable emotional, financial, and psychological strain [14,15,16,17]. For many caregivers, these challenges culminate in decisions about how and when their companion animal’s life should end.

Within high-income countries, euthanasia is the most common EOL pathway for companion animals [18,19,20,21]. While contemporary veterinary guidelines emphasize providing a rapid, painless, and distress-free death [22], pet caregivers’ EOL experiences extend well beyond the technical aspects of the procedure. Research increasingly suggests that owners’ perceptions of a “good death” are shaped by communication with the veterinary team, opportunities for preparation, emotional support, involvement in decision making, and confidence in the chosen course of action [23,24,25,26,27,28,29].

Veterinary communication appears particularly influential throughout the EOL experience. Compassionate, proactive communication has been associated with greater preparedness, increased satisfaction, and reduced decisional conflict, whereas delayed or crisis-driven communication may result in urgent, nondeliberative conversations and may contribute to anxiety, uncertainty, and lingering regret [17,30,31,32]. While previous quantitative studies have identified communication as an important predictor of caregiver emotional outcomes, they offer limited insight into the processes through which caregivers interpret EOL experiences or the meanings they ultimately assign to them [9,25,33].

Qualitative inquiry offers an opportunity to move beyond measuring outcomes to understanding caregivers’ lived experiences. It allows researchers to explore how caregivers make sense of uncertainty, negotiate difficult decisions with family members and veterinary professionals, reconcile feelings of regret, construct meaning following loss, and identify the forms of support that are most helpful. These insights can inform veterinary communication training, strengthen EOL care practices, and help veterinary teams better support pet caregivers before, during, and after a companion animal’s death.

To better understand these experiences from caregivers’ perspectives, the present study reports the qualitative phase of an explanatory sequential mixed-methods investigation of companion animal EOL experiences. Building on findings from the quantitative survey [34], this study uses reflexive thematic analysis [35,36] to explore bereaved pet caregivers’ narratives regarding the most challenging and meaningful aspects of their pet’s EOL experience, the regrets they carry following their pet’s death, and the advice they would offer veterinarians to best support families during EOL care. The findings are interpreted through the lens of three complementary theoretical perspectives: (1) stress-and-coping theory, which holds that uncertainty and limited perceived control intensify distress and shape how caregivers respond to EOL demands [37,38]; (2) shared decision-making models, which predict that communication quality shapes caregivers’ sense of agency and alignment with their own values [39,40]; (3) and continuing bonds theory, which suggests that how death is anticipated, discussed, and enacted influences how caregivers integrate loss and maintain an ongoing relationship with the deceased [41,42]. Together, these perspectives provide a framework for interpreting how veterinary communication and support influence caregivers’ emotional experiences and meaning making throughout the EOL journey.

## 2. Materials and Methods

This qualitative study utilized reflexive thematic analysis [35,36] as a framework for approaching the research process from a critical and reflexive lens. This method invites research teams to address personal biases and positionalities throughout the research process and to be intentional in acknowledging their role and influence in how they interpret and present participants’ data [43]. As such, we began the research process by determining what personal and professional roles we bring to the study. Each of the authors reflected on their clinical practice and qualitative methodological training in human–animal interactions and how our combined professional degrees in clinical sciences, public health, social work, and veterinary medicine may blind us or cloud our judgment in interpreting participants’ experiences.

## 3. Sample and Recruitment

The study inclusion criteria included adults aged 18 years or older who lived in the United States (US) and had made EOL decisions for at least one dog or cat in the past two years. A total of 802 respondents were recruited between September and December 2025. Potential participants were invited to participate through emails distributed by three large veterinary organizations providing end-of-life care services: Lap of Love, Caring Pathways, and BluePearl Pet Hospice. After following the study link to the online consent form, potential participants received information about the study expectations and incentives, and then digitally signed the consent form before accessing the study survey items. We utilized a random number generator process to select 25% of participants who provided open-ended survey responses, resulting in a sample of 143 participants for qualitative analysis. Selection was independent of participant characteristics or response content. Before the recruitment process, we obtained ethical approval from Colorado State University’s Institutional Review Board, protocol # 7297.

## 4. Data Collection

This study is the second phase of a sequential cross-sectional mixed-methods design [44] utilizing an anonymous online survey via Qualtrics (Qualtrics, Inc., Provo, UT, USA) to explore US pet caregivers’ experiences with companion animal EOL decision-making, veterinary communication, perceived preparedness, and post-loss outcomes. The quantitative survey [34] gathered pet caregiver and animal demographics (e.g., caregiver age, gender, species of pet); and the pet caregiver’s number and type of previous pet EOL experiences; the perceived quality of how their veterinary team communicated during EOL decision-making; overall satisfaction with their pet’s EOL experience and veterinary team support; perceived burden during the pet’s EOL; perception of their pet’s quality of life before death; access to quality-of-life (QOL) assessment tools; and EOL decisional regret.

The survey’s open-ended questions, the focus of this study, included the following four open-ended prompts:

- Please share the most challenging aspect for you of your pet’s EOL experience.
- What was the most meaningful aspect for you of your pet’s EOL experience?
- Please share any regrets you have about your pet’s EOL experience.
- If you could share one piece of advice with veterinarians about how to best support pet owners in preparing for their pet’s final moments, what advice would you offer?

## 5. Data Analysis

We integrated [1,2] six phases of reflexive thematic analysis as our coding team of three (second-fourth authors) approached data analysis. Since we have worked on coding projects together in the past and had familiarity with each other’s backgrounds and analytic styles, we started our first meeting by talking about how these participants’ experiences might vary from our previous samples’ experiences and how we feel our own positionalities may come into play during this data analysis project. Then we discussed our overall analysis plan and how we intended to break the work into individual and group-based parts.

We began analysis by individually reading the corpus of data and gaining an overall understanding of pet caregivers’ EOL decision-making and euthanasia experiences. Next, we individually reviewed and coded one-third of the responses to the first question (most challenging aspects). As a team, we met to discuss our coding processes, reviewed the codes, and determined some initial codes for the first question. Then, we individually coded the remainder of the first question and met as a team to review our codes, determine code names and definitions, and decide when to apply these codes. We continued the same process for the remaining open-ended questions, noting new codes as we went along. Our final meeting included reviewing the codes, developing an overall thematic structure, selecting representative quotes, and determining the format for our findings section. To protect participant confidentiality, we referenced all participant quotes with their study number (i.e., P01). Lastly, we discussed the implications of these findings for veterinary teams and how they can partner with pet caregivers to facilitate the most peaceful EOL experience possible for their pets while fostering transparent, compassionate communication that validates caregivers’ experiences and fosters meaning throughout their pet’s EOL journey.

## 6. Trustworthiness

We intentionally integrated several strategies throughout the study to enhance the trustworthiness of our findings. Working as a coding team of three allowed us to bring our unique backgrounds to our work and offer diverse viewpoints when interpreting the data. We kept audit trails to identify coding decisions such as code names and meanings, and a table to document our iterative steps in developing a thematic structure to represent participants’ experience. Additionally, the lead author of the quantitative study served as a co-lead author on this study and helped integrate quantitative findings and qualitative findings to provide context and inform practice implications.

## 7. Results

Participant demographics are reported in the parent survey [34]. Selected characteristics are summarized here to provide context for caregivers’ EOL decision-making and experiences. In the parent study, participants were predominantly middle-aged to older adults, with 64.5% aged 35 to 64, most of whom identified as women (83.3%); dogs were the most commonly reported species (72.7%). The majority of caregivers had prior experience making EOL decisions for at least one companion animal (77.9%). Most pets died via euthanasia (94.4%), most commonly through in-home euthanasia without prior hospice support (47.8%). Nearly one-third of caregivers (30.2%) reported feeling rushed in their decision-making, with 17.3% having less than 24 h to make an EOL decision. Most caregivers rated the overall quality of their EOL communication as good or very good (86.0%), with more than four in five agreeing that their veterinary team listened to their concerns, presented information clearly, and included them in decision-making. While 83.7% of caregivers reported being satisfied with their pet’s overall EOL experience, regret was reported by 34.8% of respondents.

Pet caregivers’ narratives reflect the tensions they hold in wanting to provide the most compassionate death for their cat or dog and their own desires to hold onto their pet as long as possible. Many recounted uncertainties about when euthanasia was appropriate, difficulty obtaining clear and actionable guidance from their veterinary team, and challenges preparing for their pet’s death. Alongside accounts of grief and regret, caregivers also described moments of meaning, peace, and reassurance as they reflected on their overall experiences. Many offered advice for veterinarians on how to communicate about their pet’s illness, including clear, actionable, and compassionate guidance. The findings presented below synthesize participants’ responses across prompts to provide an integrated understanding of caregivers’ EOL experiences.

The findings of this study reveal the many ways that pet caregivers experience their pet’s EOL, from the profound responsibility of knowing when it is time to euthanize, to the pain and grief of navigating life without their beloved pet, and finally to how they found or did not find meaning within the experience.

The most prominent challenge described by caregivers was determining when it was “time” to proceed with euthanasia. Participants repeatedly emphasized the uncertainty of making this decision in the absence of a clearly identifiable “right” time. This uncertainty stemmed from a range of circumstances, including ambiguous prognoses, gradual declines in health, and situations in which a pet experienced a sudden accident or rapid deterioration, leaving caregivers with little or no opportunity to make an EOL decision. These situations were often compounded by disagreements among family members regarding the appropriate timing of euthanasia and by the emotional and physical demands of caregiving. In addition, pet caregivers frequently communicated feelings of regret or guilt around EOL care, particularly regarding the timing of euthanasia, available treatment options, poor interactions with veterinary teams, and the setting in which the euthanasia occurred.

When pets experienced more gradual declines, caregivers commonly partnered with veterinary teams to develop end-of-life care plans for their pets. Veterinary teams’ compassionate and transparent communication about the animal’s life expectancy and quality of life provided caregivers an opening to be creative in how they curated their pet’s end of life. Pet caregivers reflected on meaningful final experiences, such as spoiling their companion animal during the final days or weeks or gathering family and friends to say goodbye.

Throughout the open-ended questions, caregivers described aspects of veterinary care that either fostered a sense of support throughout their companion animal’s EOL experience or created barriers to informed, compassionate decision-making. Responses to the final open-ended question provided an opportunity for caregivers to translate their experiences into recommendations for veterinary professionals. Their responses provided deeper insight into how caregivers experienced companion animal EOL care and highlighted opportunities for veterinary professionals to better support families before, during, and after a pet’s death.

### 7.1. The Tensions in Finding the “Perfect Time”

Reflecting on their pet’s EOL decision-making, caregivers described navigating complex emotional and practical tensions as they sought to balance their companion animal’s quality of life, their family’s needs, and their own hopes, fears, and readiness to say goodbye. Pet caregivers portrayed their animal as an essential member of their family and relayed the immense stress they felt as the ultimate decision-maker about when and how the dying process would occur. Many caregivers described the emotional distress experienced by “letting go of someone so woven into my life and being” (P59). These tensions commonly centered on obtaining enough information to make an informed decision about the timing of euthanasia, balancing the desire to minimize their companion animal’s suffering with the hope of preserving as much meaningful time together as possible. One pet caregiver explained this tension as “Knowing when is the right time [and] finding the sweet spot between waiting too long and doing it too soon” (P77). Many caregivers described struggling to say goodbye, not only because of the finality of the decision but also because they anticipated the profound absence their companion animal would leave in their daily lives. One caregiver explained:

Not knowing how to say goodbye to my best friend. I couldn’t grasp that one day that was fast approaching I would see that small precious powdered sugar face looking back at me for the last time. Sometimes when he was sleeping, I would just stare at him with tears streaming down my face, and I would ask him ‘How do I prepare to say goodbye to you? How can I accept that one day I will wake up and never see you again?’ It was the hardest and most painful experience for me to this day (P58).

Factors influencing the timing of euthanasia included caregivers’ access to information about their pet’s health and prognosis, loss of mobility, visible pain or suffering, family dynamics, and a perception that the pursuit of aggressive medical care would be futile. For some, however, the decision became clearer as their pet experienced a rapid decline in health, leaving little doubt that euthanasia was the most compassionate option. One caregiver reflected on “Watching this beautiful girl go from happy and healthy to lethargic and losing her life despite her spirit” (P132). The sudden need to make EOL decisions was particularly difficult for caregivers whose pets experienced an unexpected health crisis or whose previously undiagnosed conditions rapidly progressed, leaving little time to prepare. Expressions of helplessness and hopelessness emerged across responses, such as a pet caregiver’s experience in “feel[ing] like it was a lose-lose situation. I wonder if I should have had him euthanized or should have gone to the vet yet again with him, but everything felt so hopeless” (P24). Although less common, a small number of caregivers described the anguish associated with behavioral euthanasia, reflecting on the emotional burden of deciding whether euthanasia represented the most compassionate course of action for their companion animal.

For caregivers who faced ongoing uncertainty about their pet’s prognosis, determining the timing of euthanasia while hoping to give their companion animal the best possible final days was especially challenging. One caregiver reflected, “It was very difficult as our dog seemed improved and so very happy the day we did the euthanasia. I had to physically restrain him, and he clearly knew what we were doing, and he was not ready to go. That part was heartbreaking” (P112). Similarly, a pet caregiver described their biggest challenge as “not knowing for sure she would not get better given more time and yet knowing more time would add to her suffering” (P28). Many caregivers also described the physical and practical demands of caring for a companion animal with a progressive illness. Participants frequently reflected on the cumulative toll of caregiving, including “witnessing his slow decline [and the] caregiver fatigue as he required more and more assistance” (P88). Changes in their pet’s status weighed heavily on caregivers’ emotional energy, as one day could bring hope with small improvements and the next, dreaded fears that accompany a presumably imminent death.

Family dynamics also contributed to the complexity of EOL decision-making. Caregivers frequently described disagreements about the timing of euthanasia, with some family members believing it was time to say goodbye while others felt it was too early to consider euthanasia. These differing perspectives often left one family member carrying the primary responsibility for making the final decision while simultaneously confronting the reality of their companion animal’s declining condition. As one caregiver explained, “[My] spouse wasn’t ready, but [our] pet was suffering. He just couldn’t say goodbye until it was clear [that our] pet would die naturally and painfully within 2–3 days” (P103). Similar tensions emerged among caregivers with children, who described balancing their own grief and decision-making responsibilities with supporting their children’s emotional needs. Balancing the needs of self-versus others appeared as follows:

My children’s ability to process the loss of a pet that has been with them their whole entire life. Also, in the aftermath—I have twin children. They ‘took turns’ of who got to keep the paw print and lock of hair in their room at night. This was really hard because I was trying to help them process grief, and my own at the same time. I also chose to not let the kids be present for the home euthanasia because I thought it would be too difficult for them to understand that we couldn’t just fix our pet. But they also knew he was ready to pass (P137).

In summary, pet caregivers revealed emotional pressure surrounding the timing of EOL decisions as they sought to balance minimizing their companion animal’s suffering with their desire to extend their time together.

### 7.2. Living with Regret and Guilt

Reflecting on their companion animal’s EOL experience, many caregivers described feelings of regret. Consistent with the uncertainty surrounding EOL decision-making, these regrets often centered on the timing of euthanasia. Other sources of regret included treatment decisions, interactions with the veterinary team, and the setting where their companion animal died. Questions weighing on one pet caregiver’s mind were: “If [I] had bought more time, if [I] had chosen hospice and had access to supportive meds” (P8). For some caregivers, even a matter of hours became a source of lasting regret:

My only regret was choosing a noon appointment for her euthanasia and not the earlier 9 am slot, because I saw how much those extra hours took out of her, but honestly, the entire process was so sudden that *I* needed that extra time with her (P38).

Regret was frequently accompanied by feelings of guilt. Caregivers often questioned whether being overwhelmed by the emotional demands of the EOL experience or by the strength of their bond with their companion animal had influenced the decisions they made. As one pet caregiver explained, “I just felt overwhelmed and that he wasn’t ready, but I didn’t want him to suffer. I have guilt, and I miss him.” (P28), while another shared how their “emotional attachment made it hard to make EOL decisions. The guilt I have for making that decision, even though I know it was the right thing to do” (P9). Another caregiver described how the final moments of their companion animal’s death continued to evoke feelings of guilt:

I wish we could have done it at home, and a few hours sooner. And I don’t know that we could have changed anything around how he reacted while the drug was being pushed, but it made his last moments SEEM less than peaceful to us, and I’ve never been able to shake that image from my mind (P42).

Although regret was common, a small number of caregivers reflected on how time helped them gain perspective and recognize that they had made the best decision possible with the information available at the time. As one caregiver described, they reconciled their “ethical qualms and feel[ing] more confident that euthanasia can mean assisting a loved one in choosing death themselves, not choosing it ‘for them’” (P79). Together, these narratives illustrate that caregivers’ reflections on EOL decisions continued long after their companion animal’s death, with many continuing to grapple with regret and guilt while others gradually found reassurance and acceptance.

### 7.3. Caregiver’s Meaning-Making Processes

#### 7.3.1. “She Knew It Was Time”: Pet Caregivers’ Perceptions of Their Animal’s Signaling Behaviors

Many caregivers described finding comfort in perceiving that their companion animal had accepted the EOL decision. Participants often interpreted their pet’s behavior as signaling readiness, peace, or an awareness that it was time to let go. As one caregiver explained, their pet “knew it was time and accepted it. She was tired, and we all knew it. She was ok with it and so were we” (P64). Similarly, another pet caregiver explained, “It was almost like she knew what was happening—and welcomed it” (P38). Another reflected on how their pet held pride in not showing their physical suffering and how they “tried until the end” (P5). Conversely, one caregiver found relief when they felt that they were ending their pet’s “sense of sadness over not “doing his job” (P10). Some caregivers also described finding comfort in perceived signs from the afterlife. These welcomed messages were sought by one pet caregiver who “asked my dog to give me a sign of her passing,” and who later received a sign of not just “a cardinal, but a large flock of cardinals showed up” (P114). For these caregivers, such experiences provided comfort and represented an important step in finding meaning and maintaining a sense of connection following their companion animal’s death.

#### 7.3.2. “A Heartbreaking but Meaningful Day”

Caregivers’ narratives highlighted the meaning-making processes that unfolded both before and after their companion animal’s death, including the importance of intentionally planning their companion animal’s final days. They described how creating the best possible final moments provided comfort, facilitated closure, and helped them begin to make meaning of the loss. Although many participants characterized their companion animal’s final moments as “beautiful,” “peaceful,” or “calm,” others viewed the experience as emotionally painful and traumatic. One caregiver, reflecting on the last day, simply stated: “None. It was a horrible day, and our hearts were broken” (P15). Together, these narratives illustrate the diverse ways caregivers interpret and find meaning in their companion animal’s EOL experience.

For caregivers who found meaning in the grieving process, intentionally creating positive final experiences with their companion animal brought comfort to both them and their loved ones. They described taking comfort in spending time together outdoors, offering favorite foods that had previously been reserved or restricted, and ensuring their companion animal’s final moments were peaceful and surrounded by love. One caregiver described the setting they thoughtfully created for their pet:

My dog loved ginger snap cookies, so we sat in our living room on the floor and let him eat as many cookies as he wanted. We were relaxed, and he was happy! After about an hour of petting him and us all talking, the vet administered a drug to help him relax. Then another drug to put him to sleep before the final drug. I can’t think of a better way it could have gone for all of us (P53).

Having time to plan their pet’s final moments allowed for creativity in what their beloved animal’s final moments could entail. Several commented on how it felt meaningful to spoil their pet in their final moments. A family that mobilized the support of hospice explained:

The hospice experience was extraordinary. Since we scheduled it in advance, we got our boy ALL the forbidden snacks to eat before he crossed over. Chocolate milkshake, Reese’s, steak, fries, so many snacks. We watched his favorite Bluey episode. In the months leading up to it, we treated every day like it was the last. We didn’t get frustrated, we didn’t feel irritated, we just loved him for all that he was. It really made a difference (P16).

Another family described working closely with their veterinarian to develop an EOL plan that minimized their dog’s distress while allowing family and friends time to gather, share their love, and say goodbye before the veterinarian arrived at their home:

We had all of our friends and family come to a goodbye party for [our dog] in the morning before the vet came in the afternoon for his in-home euthanasia appointment. He got to spend the first half of his very last day literally as the center of attention, eating a hamburger and doggy ice cream, surrounded by his favorite people. It truly felt like he knew everyone was there to say goodbye, but that he was truly happy and at peace. When the time came, everyone left so that [he] could spend his final hour with just us. It was a heartbreaking but beautiful and meaningful day—and it would not have been possible without the option of in-home euthanasia (P138).

Many caregivers described taking comfort in remaining physically close to their companion animal during their final moments, with these experiences becoming cherished memories following the loss:

I loved that I was able to hold [him] in my arms and comfort him as he left this world in a peaceful, relaxed, and comfortable way. For many years, he sat next to me on the couch as I worked from home on the laptop. To sit in that same spot, with him in my arms—Very, very special (P126).

Stories also highlighted little things that pet caregivers/families encountered that brought them peace: “Oddly enough, the most meaningful part was hearing him snore when he was under sedation. He hadn’t slept peacefully in months” (P52).

Interwoven throughout responses were pet caregivers’ acknowledgments of how their veterinary team’s guidance and medical support enabled them to plan for their pet’s peaceful passing.

#### 7.3.3. Support with Sacred Life on the Line

Despite the profound grief associated with losing a companion animal, many participants expressed sincere gratitude for the compassion, communication, and support provided by their veterinary team throughout the EOL process. One participant said:

Gauging the suffering of an animal that hides pain from you is so hard. We need all the support possible from vets and care teams to know when to assist with death, if at all. Making decisions when sacred life is on the line is something that should be done with humility and deep respect, so I am grateful when vets and care teams show that respect (P83).

Frequently, pet caregivers indicated that it was meaningful when veterinary teams took the time to provide a clear, compassionate description of euthanasia. One participant described their veterinarian’s communication as “very clear and compassionate. Gentle is a good word to describe it” (P66). Other participants noted their veterinarian was “extremely respectful, kind, patient, and communicated every step of the process clearly” (P94), in addition to having “empathy for the humans and gentle care for our dog” (P118). Caregivers frequently described clear and compassionate communication as instrumental in helping them feel at peace with the decision to proceed with euthanasia, reinforcing that they were acting in their companion animal’s best interest by alleviating suffering. One pet caregiver said, “Knowing my dog was with me and her favorite people in her home when she passed, my voice was the last thing she heard when she passed on. The vet who was with us, reassuring us and letting us know that my dog was in discomfort, the way she would close her eyes because she was in pain from the tumor, and knowing it was the right time” (P54).

Many caregivers expressed appreciation for memorial keepsakes provided by their veterinary team, such as paw prints and locks of fur: “A hospital staff member made me ink paw prints, and I clipped off my favorite sections of his coat as keepsakes” (P48). Pet caregivers also really appreciated friends sending cards, and said things such as:

I was really touched when his emergency vet provider sent a lovely book with photos of him included in the book. I’d send updates over the past year to them with photos, and they took the time to print the photos out and put them in the book along with written messages. I got it in the mail and just started bawling when I opened up the envelope. It was so thoughtful. It was really touching. It also made me feel less alone (P115).

Some respondents discussed the importance of having their pets’ primary care veterinarian, or a veterinarian who knew the animal well, support the euthanasia and decision-making process. They described this continuity of care and established relationship as a source of trust, reassurance, and comfort during an emotionally difficult time. One participant explained, “We had a really good vet team who had gotten to know her and were able to objectively help with the decision, as I had a lot of guilt about it” (P93). While another mentioned:

We have been with our family vet’s practice for 20 years. They know us, and we know them. We knew that they loved her, too, and their support was priceless. Also, the vet who came to the house from [a home euthanasia provider] was gentle, kind, and respectful. There was no rush. Every action was explained, and we were asked if we were ready for each step. “I don’t hear a heartbeat” were gentle words to hear rather than a bare pronunciation of death (P100).

Some caregivers expressed disappointment that their primary care veterinarian was unable to participate in the euthanasia because of extenuating circumstances, wishing that the veterinarian who had cared for their companion animal over the years could have been present during the final moments. “Wish we would have been able to have his primary vet there; she was on maternity leave and didn’t get to say goodbye in person to him. But the other vets did an awesome job taking care of us, and she did call in to the office and speak to us on the phone right before he was euthanized and told us she was there with us in spirit” (P39). For many caregivers, support from a familiar veterinarian provided reassurance and helped alleviate the emotional burden of quality-of-life assessments and EOL decision-making.

#### 7.3.4. When Support Falls Short: Barriers and Burdens

Although many caregivers described receiving exceptional support from their veterinary team, others wished they had received more guidance, information, and emotional support throughout the EOL process. Participants also described barriers to accessing veterinary care, particularly in-home euthanasia services, with several expressing that they were either unaware that these services existed or unable to access them when needed. One caregiver reflected on these challenges, stating: “Tests were not able to be done ahead to know for all that was going on. [Being from a] rural area and a test I wanted done would have meant driving 6 h one way with the very sick dog traveling to the specialist” (P22).

Other participants reported feeling emotionally unsupported while they were receiving medical care: “Being alone and feeling like nobody at the ER cared about me or what I was going through because nobody checked in with me” (P98). Another participant voiced their concerns about the lack of support from their primary care veterinarian: “My vet was no help. They only wanted to run expensive tests. My senior diabetic dog, in liver failure, started having seizures. My vet friend delicately discussed the reality of her situation, and I was able to make decisions to honor her and end her suffering. My vet only wanted to run expensive tests and never broached the subject of quality of life” (P97). In addition to unmet emotional support needs, caregivers also described the substantial financial burden associated with emergency and EOL veterinary care. Many reflected on the difficulty of facing significant treatment costs while ultimately deciding that euthanasia was the most compassionate option. One participant stated, “$14 k spent on chemo that probably bought him no additional time was unfortunate. Nothing the vet team could have done differently, just life being life” (P1).

Beyond medical and emotional challenges, caregivers encountered several logistical barriers during their companion animal’s final hours. Participants described loved ones being unable to reach the veterinary hospital in time, weather delaying access to care, facing euthanasia without family or friends present, and feeling that the opportunity to say goodbye was cut short. One pet caregiver shared, “I wish I would’ve gotten more time to say goodbye that day, and I wish he could’ve seen me and not been facing away from me. I also wish I would’ve felt more confident in the decision we made to help him [find] peace at that time.” (P49). Several caregivers described being surprised by how quickly their companion animal died after the euthanasia injection, leaving them wishing they had just a few more conscious moments together. These reflections underscored the importance participants placed on having sufficient time to say goodbye before the euthanasia procedure began.

After their pets’ passing, caregivers described struggling with both the emotional absence left by their pets and the lasting memories of witnessing the euthanasia or death, particularly when it occurred at home. One pet caregiver explained, “It was at home, so always remembering that was the spot she passed away” (P12). Another said, “Learning to live without him in my home when a piece of my heart went with him” (P15). Pet-related grief is common, and many pet caregivers have difficulty after their pet passes. This challenge was described by a participant: “Letting him go. He was the light of my life and my heart and soul. He was special needs, and we were so close. I’m having a really hard time with him being gone” (P24).

### 7.4. Recommendations for Veterinary Teams

The findings of this study highlight avenues for veterinary teams to help pet caregivers feel supported, confident, and informed in their EOL decisions for their pets. They also suggest that compassionate communication, thoughtful preparation, and individualized support may foster caregivers’ ability to find meaning and peace following their companion animal’s death.

Notably, participants rarely identified the technical or medical aspects of EOL care as sources of challenge or regret, nor did their recommendations for veterinary professionals focus on medical management. Instead, caregivers consistently emphasized the importance of clear, compassionate, and timely communication about their companion animal’s condition, prognosis, and EOL options as central to feeling supported and finding peace with the decisions they ultimately made. Pet caregivers also conveyed that alongside support and communication, emotional connection with the veterinary team was deeply valued. Suggestions for compassion, empathy, and patience were woven through each theme within the question of what advice they would like veterinary providers to know. As one pet caregiver succinctly stated, “Be kind, be honest, and be patient” (P108).

Another pet caregiver stated, “…be more compassionate. My loss before this recent one was at an ER vet clinic, and the vet didn’t even say anything except to give me choices. After that, it was a tech that gave my baby his last injection” (P16). This account underscores that, for many caregivers, high-quality EOL care extends beyond informed consent and clinical decision-making to include empathy and emotional presence.

Consistent with the findings from the quantitative study [3], caregivers emphasized the importance of veterinary professionals initiating EOL conversations early and engaging in honest, transparent discussions about diagnosis, prognosis, and what to expect as their companion animal’s condition progresses. One pet caregiver described how their veterinary team helped support them through the hospice process:

There are so many positive things I could say about my vet, techs, and vet specialists. What was incredibly important to me and appreciated was how well my vet communicated with me. There were a lot of phone calls and emails back and forth to answer questions, concerns, changes in his status, etc. She was always open to discussion, assuaged fears, provided support and guidance, and never made me feel like an inconvenience. I had all the information I needed to make the best decision for my dog without any uncertainty or regrets. That allowed me to really enjoy our final months spent together (P17).

However, many caregivers described not receiving this level of early, clear communication regarding their companion animal’s condition. Many perceived that veterinary professionals were hesitant to initiate or engage in conversations about prognosis and EOL planning. Two pet caregivers used the phrase, “beating around the bush” (P49, P55) when describing EOL communication from their veterinary team, another sharing, “Say the hard parts out loud. We need to hear it” (P28). Several caregivers described perceiving this hesitation as contributing to delayed euthanasia decisions and, ultimately, lingering regret. As one caregiver described, “I felt like they were afraid to tell me the blunt truth and maybe should have done that the week before when we were there” (P104).

Many caregivers lamented both a lack of awareness of in-home hospice and other EOL care services and limited access to these options when they were available. One caregiver, despite describing a strong emotional connection with their veterinary team, expressed disappointment that hospice care had never been presented as an option:

I feel like our vet clinic did everything right that day and were very comforting. They cried right along with us, and we knew how much they loved him, too, as he had been one of their frequent [patients] for years. I do wish I had known about and had hospice support; it was mentioned only after we made our decision, and we didn’t want him to suffer anymore at that point (P19).

Many caregivers also expressed a desire for practical resources, presented before euthanasia, to help them navigate the EOL journey, including grief resources, quality-of-life assessment tools, support groups, ideas for creating meaningful final experiences with their companion animal, and information about memorial options. Notably, participants suggested that simply being provided with these resources could be valuable, even if time constraints prevented veterinary teams from discussing each option in detail. As one caregiver explained:

Point people to resources if they can’t take the time themselves. Refer them to a pet death doula or support groups. Give them a website that has EOL guidance from quality of life calculators to bucket list ideas and after-death options. If you can’t take the time to go over everything with them, make sure they know where they can go to get that (P36).

Participants consistently described wanting more anticipatory guidance before the euthanasia appointment. A pet caregiver describes being unprepared and uncertain of what was happening during the euthanasia: “Let them know what the pet is able to see, hear, and feel at each step of the euthanasia process. I kept petting my cat but realized she probably couldn’t feel anything at that point but maybe I should be talking to her instead so she could hear my voice. More guidance like that would be important” (P98). Further, pet caregivers wanted ample time to say goodbye during the euthanasia process, as a pet caregiver clearly stated, “Give the pet owner plenty of time to say goodbye before euthanasia is done” (P81).

Many pet caregivers just wanted to be heard and reassured in their decision. A pet caregiver described this as “Have compassion for pet and owner. Listen to owner[s’] feelings. Explain clearly any symptoms. Support decision” (P6). Another succinctly stated, “Just listen” (P13).

In addition, caregivers emphasized that high-quality EOL care was a team effort, valuing both the clinical expertise and emotional support provided by veterinarians, technicians, assistants, and other members of the veterinary team. A pet caregiver offered, “Utilize the team. The veterinary staff often have more time to talk to clients and, at times, can have a closer connection with the pet. Designating someone to call after a difficult diagnosis to go over EOL options would have been really nice” (P45). Another expanded, “Everything you do matters, and small personal touches (a truly handwritten card, for example) mean a lot” (P92).

## 8. Discussion

Findings from this study advance our understanding of how caregivers experience, interpret, and make meaning of companion animal EOL care, including the challenges they face, the regrets they carry, and the role veterinary teams play in shaping these experiences. Although caregivers’ narratives centered on the immense responsibility of making decisions surrounding their companion animal’s death, many also described finding meaning and peace throughout the EOL journey. Instrumental to the meaning-making process was the ability of pet caregivers to know they had done the best they could to minimize their pet’s distress [4]. Participants found comfort in being able to prepare for their companion animal’s final moments, creating opportunities for connection, pampering, and peace. Conversely, regret was most often associated with feeling unprepared, witnessing perceived suffering, or feeling they had insufficient time to say goodbye [5]. Situated amid these multifaceted decision-making processes were deeply bonded relationships in which pet caregivers navigated decisions they portrayed as unbearable, untimely, and inadequately prepared for [6,7]. The following section highlights the study’s key findings and considers how veterinary teams can better prepare families through earlier communication, proactive EOL planning, and compassionate support before, during, and after a companion animal’s death.

### 8.1. Key Findings and Recommendations

A central source of distress for caregivers was uncertainty regarding whether additional medical treatment could meaningfully improve their companion animal’s condition or whether it was time to prioritize a peaceful death. When outcomes were clear, pet caregivers appeared to hold less guilt about their decision for expedited euthanasia [3]. Scenarios with greater uncertainty placed pet caregivers in a difficult position of choosing between their own needs and their pet’s best interests. The ability to hasten their pet’s death and reduce suffering was portrayed as both a blessing and a curse. Many caregivers described lingering doubts about the decisions they made, yet they also reflected on doing the best they could with the information and circumstances available at the time. Although behavioral euthanasia was described by only a small number of caregivers in the present study, their narratives highlighted the emotional burden they experience with these decisions, supporting research on behavioral euthanasia that suggests caregivers often struggle with decision-making when serious behavioral concerns create competing interests related to animal welfare, human safety, and the human–animal bond [8]. The psychosocial experiences and bereavement outcomes of caregivers following behavioral euthanasia remain understudied and represent an important area for future research.

Their narratives suggest that the search for a “right” decision often gave way to the realization that there is rarely a perfect way to say goodbye to a beloved companion animal [4]. Our findings align with previous research documenting the profound grief experienced by bereaved pet caregivers [9,10,11,12]. Extending this work, participants in the present study described how grief often remained intertwined with enduring feelings of regret and guilt regarding EOL decisions. Given that pet caregivers commonly grieve in private [13], it is imperative that systemic processes are in place for veterinary teams to support clients early in their decision-making processes to open opportunities for expression of grief.

With time, many caregivers described finding meaning by focusing on the loving actions they took during their companion animal’s final days. Their narratives emphasized intentional acts of connection, comfort, and preparation, suggesting that these experiences became enduring sources of peace as they reflected on the loss. Creating meaningful final experiences, such as offering favorite foods, surrounding their companion animal with family and friends, engaging in familiar routines, and providing a peaceful setting for their final moments, appeared to bring comfort to many caregivers [7]. Honoring the needs of bereaved family members and pets in the home was an additional consideration that pet caregivers felt compelled to address. Opportunities to thoughtfully plan these meaningful rituals were often attributed to transparent communication from trusted veterinary providers who knew the family well or who facilitated referrals to trusted in-home euthanasia services [4,14]. Signs from their pets, either before and/or after death, provided comfort for many caregivers and served as continuing bonds that helped them navigate their grief [15,16].

### 8.2. Implications

Guided by caregivers’ reflections, our findings suggest several practical implications for veterinary practices. We encourage veterinary teams to initiate EOL conversations early and revisit them throughout the course of chronic or progressive illness, allowing caregivers to discuss their communication preferences, goals for care, and perceptions of an acceptable quality of life for their companion animal. These conversations should also explore how much information caregivers would like to know about their pet’s health status, how involved they want to be in decision-making processes, and their preferences regarding aggressive treatment, palliative care, or a combination of both. Veterinary teams should be mindful of how they address financial options and the amount of testing and life-extending procedures pet caregivers would like to explore. Providing a range of options and cost points with transparency about the benefits and limitations of each option can help pet guardians discern which plan of care might be best for their pet. Introducing these conversations before a companion animal enters a health crisis may reduce the stress of emergency EOL decision-making by helping veterinary teams better understand caregivers’ goals, decision-making preferences, and desired timing and depth of EOL discussions [7].

Veterinary teams should begin by assessing caregivers’ understanding of their companion animal’s physical comfort, emotional well-being, and quality of life [14]. These conversations provide an opportunity to tailor education, decision-support tools, and other resources to caregivers’ individual informational needs and their confidence in recognizing changes in their companion animal’s condition [14]. As a companion animal’s health declines, veterinary providers should invite pet caregivers into conversations about planning for their pet’s best last days. These conversations should be tailored to the pet’s condition, the pet caregiver’s interests, and their access to services and resources. For those who may be afforded time to plan for their pet’s death, veterinary teams can invite pet caregivers to consider planning for their pets’ final moments and how they can create rituals around their pet’s favorite food, location in the home, people in their lives, and favorite place to sleep.

Our study found that many caregivers wished that conversations about quality of life and EOL options were discussed earlier and in more depth during their pets’ care and treatment. The quantitative data from this study found that when EOL conversations were initiated after the pet’s condition significantly worsened, or during a crisis, 38% of participants felt the conversation was too late [3]. These findings appear similar to a study in which more than one in five pet caregivers felt that discussions about EOL occurred too late [17]. Recommendations for earlier EOL conversations are supported by findings from both the present study and previous research. In our companion quantitative study, most caregivers reported that EOL conversations were initiated by themselves or family members rather than by the veterinary team [3], suggesting a desire for earlier conversations. The importance of proactive EOL discussions is further supported by previous research reporting that more than 80% of caregivers perceived palliative care, focused on maximizing comfort rather than prolonging life, to have positively affected their companion animal’s quality of life during the final stages of life [17]. Such care, however, depends on timely conversations about goals of care, quality of life, and available EOL options [17]. Likewise, other studies have shown that many caregivers wish to plan well in advance of their companion animal’s death. For example, 28% of owners indicated they prefer to finalize all EOL decisions far in advance of their pets’ death, underscoring the importance of veterinarians proactively discussing the full range of EOL options [18,19].

Importantly, our findings also reinforce that there is no perfect approach to EOL communication. Consistent with previous research, caregivers described diverse informational and emotional support needs throughout the EOL process [14]. Many participants expressed a preference for shared decision-making, where their veterinarian shared information, and they made the final decision together as equal partners. This aligns with previous studies which found that the majority of pet owners preferred shared decision making during veterinary consultations [20,21,22]. Furthermore, several guides to effective communication about EOL care for veterinarians emphasize the client and veterinarian acting as collaborators throughout the process [23,24,25,26].

Beyond shared decision making, pet owners valued expressions of compassion from the veterinary team following their companion animal’s death. Participants described follow-up calls, empathy, and memorial keepsakes (e.g., paw prints, locks of fur) as meaningful gestures that acknowledged their loss and helped foster continuing bonds with their companion animal. These findings align with previous research demonstrating that memorialization can facilitate grieving and provide comfort following pet loss [27,28] and offer tangible reminders of a companion animal that promote meaning-making and an enduring sense of connection; thus helping bereaved caregivers cope with grief and emotional distress [29].

Not all pet owners were as fortunate to receive access to empathetic and communicative veterinary teams. Many participants reported lacking access to end-of-life information or services due to a lack of available appointments or because they live in rural areas. Veterinary care deserts are a well-known issue; however, few studies have addressed planning and provision of end-of-life care in rural areas [30,31,32]. The ones that do highlight the need for at-home end-of-life care and information in more remote locations through mobile and telehealth options [31,32,33]. When living in areas without access to veterinary emergency care, hospice, or even veterinary social work or mental health services, veterinarians should start dialogs early about how pet caregivers want to support their pet when health decline becomes imminent. Resources could be in the form of a pamphlet, website, referrals to support groups, or online counseling services.

While many of the practical recommendations offered by caregivers in the present study echoed findings from the companion quantitative investigation [3], the qualitative narratives extended these findings by revealing the importance caregivers placed on the relational aspects of care. Beyond information and decision support, participants consistently emphasized the need for empathy, authentic emotional connection, compassion, patience, and feeling genuinely cared for by their veterinary team throughout the EOL journey. This finding aligns with previous research that places a distinction between technical “on-the-job” knowledge and “from-the-heart” care work, emphasizing the emotional, relational, and social dimensions of professional caregiving [34,35,36]. While previous research has found that veterinarians offer less intensive care labor to clients than other professional care workers [37,38], one study not only found that veterinary clients valued emotional intimacy, familiarity, and empathy but also that there was significant overlap between client–social worker and client–veterinarian relationships. The study found that both subsects of clientele had more trust in professional care workers who utilized their “from the heart” knowledge to inform their “on the job knowledge,” and had appreciation for those who applied both sets of skills when providing professional services [36]. Consistent with this framework, our findings suggest that caregivers value not only clinical expertise but also genuine human connection. However, the approach is only beneficial to professional care workers if a balance of emotional intimacy and professional rationality is achieved. Studies of nurses show that professional care workers are able to integrate this balance into their professional identities, cultivating “intimate trust” with “strategic forms of emotional control” [39,40]. In this current study, feeling seen, heard, and emotionally supported by the entire veterinary team emerged as a defining feature of a positive EOL experience. Together, these findings suggest that excellence in companion animal EOL care requires the integration of technical competence with relational and communication skills. Veterinary education and continuing professional development should therefore cultivate these competencies alongside clinical training. Other caring professions, including social work and nursing, may offer valuable models for balancing emotional intimacy with professional boundaries, providing guidance for veterinary teams seeking to deliver compassionate, relationship-centered EOL care.

## 9. Limitations

Several limitations should be considered when interpreting these findings. First, participants were recruited through an online panel and self-selected to participate, raising the possibility of selection bias. Individuals with particularly positive or negative EOL experiences may have been more likely to respond than caregivers whose experiences were less emotionally salient. Notably, participants in this sample emphasized geographic, temporal, or provider-related barriers to care rather than financial barriers, suggesting that findings may be biased toward the perspectives of individuals with the financial means or geographical proximity necessary to access euthanasia for their pets and may not represent the experiences of those without such resources. Because the sample consisted primarily of women and predominantly dog caregivers, the findings may not fully reflect the experiences of men, cat caregivers, or more diverse populations. This is particularly relevant given evidence that women, on average, report stronger attachment to companion animals and may be more likely than men to conceptualize pets as family members or children [41]. In addition, all data were retrospective and based on caregiver self-report, making them susceptible to recall bias and the influence of grief on participants’ recollections of events. Although eligibility was restricted to caregivers whose companion animal had died within the previous two years to minimize recall bias, participants’ narratives reflect their interpretations of their experiences rather than objective accounts of veterinary interactions. Lastly, as with all qualitative research, the goal was to develop a rich understanding of caregivers’ experiences rather than produce statistically generalizable findings. Nevertheless, the large sample and consistency of themes across participants provide confidence that these findings capture experiences that are likely meaningful for many bereaved companion animal caregivers.

## 10. Future Directions

The findings suggest several directions for future research. One important next step is the development and validation of a practical EOL communication preferences tool that veterinary teams could use to assess caregivers’ communication preferences, informational needs, goals of care, and perspectives regarding quality of life before a health crisis occurs. Such a tool could be introduced as companion animals age or develop life-limiting illnesses and revisited throughout the disease course to ensure that communication and care planning remain aligned with the changing needs of both the animal and the family.

Future research should also examine veterinary professionals’ perspectives on EOL communication to better understand the barriers that prevent earlier and more proactive discussions. Potential barriers may include time constraints, discomfort with emotionally difficult conversations, uncertainty regarding caregivers’ preferences, insufficient communication training, or concerns about causing distress. Understanding these barriers could inform the development of targeted educational programs, communication tools, and practice models that support veterinary teams in providing the timely, compassionate, and individualized communication caregivers consistently described as most valuable.

## 11. Conclusions

Companion animal EOL care extends far beyond medical decision-making. While caregivers described profound uncertainty, grief, and responsibility in determining when it was time to say goodbye, they also demonstrated that meaningful EOL experiences are possible when they feel informed, supported, and emotionally connected to their veterinary team. Across participants’ narratives, the defining features of positive EOL experiences were rarely technical aspects of care. Instead, caregivers consistently emphasized early and honest communication, shared decision-making, compassionate relationships, opportunities to prepare for their companion animal’s final days, and continued support following the loss.

Together, these findings suggest that excellence in veterinary EOL care requires the integration of clinical expertise with relationship-centered communication and compassionate caregiving. By initiating conversations earlier, tailoring communication to individual caregiver preferences, and supporting families before, during, and after a companion animal’s death, veterinary teams have the opportunity not only to facilitate informed decision-making but also to shape how caregivers remember one of the most significant experiences in the human–animal bond.

## Data Availability

Data are available upon request from the first author.
